# International Medical Congress

**Published:** 1893-12

**Authors:** A. Jacobi

**Affiliations:** 110 W. 34th Street, New York


### INTERNATIONAL MEDICAL CONGRESS.

The undersigned chairman of the American National Committee of the International Medical Congress, which was postponed from September 24th on account of Cholera prevailing in Italy, has been notified by the Secretary General that the Congress will be held at Rome from March 29th to April 5th, 1894. Instructions and documents relating to the journey, etc., are promised for the near future.

Yours very respectfully,

A. JACOBI, M. D.

110 W. 34th Street, New York, November 17th, 1893.

